# Supplementary material for: Clinical outcomes of chikungunya across age groups: A systematic review
Source: PLoS Negl Trop Dis. 2025 Oct 21;19(10):e0013580. doi: 10.1371/journal.pntd.0013580 (PMC12539745; doi:10.1371/journal.pntd.0013580)
Supplement: S1 File — (DOCX) [file pntd.0013580.s001.docx]

**S1 File. Search string and PICOS criteria.** The Embase and Pubmed search strategies and PICOS criteria used in this SLR.

| **String Number** | **Query** | **Hits** |
| --- | --- | --- |
| **MEDLINE** |  |  |
| 1 | “Chikungunya fever”[Mesh] OR “chikungunya”[tiab] | 7,864 |
| 2 | “case reports”[pt] OR editorial[pt] OR letter[pt] OR comment[pt] OR “clinical trial, veterinary”[pt] | 4,481,928 |
| 3 | #1 NOT #2 | 7,089 |
| 4 | #3 AND 2023/07/05:2024/09/17[dp] | 674 |
| **EMBASE** |  |  |
| 1 | ‘chikungunya’/exp OR ‘chikungunya’:ab,ti | 10862 |
| 2 | 'case study'/it OR 'case report'/it OR 'abstract report'/it OR editorial/it OR ‘veterinary clinical trial’/it OR letter/it OR note/it | 3,126,981 |
| 3 | #1 NOT #2 | 9,958 |
| 4 | #3 AND [05-07-2023]/sd | 1,087 |

| **Category** | **Inclusion criteria** | **Exclusion criteria** |
| --- | --- | --- |
| Population (P) | - Patients infected with CHIKV (including mixed populations) | None |
| Intervention (I) | Any or no intervention | None |
| Comparators (C) | Any | None |
| Outcomes (O) | - Incidence of symptomatic disease - Duration of acute illness - Duration of chronic illness - Underreporting factor - Chronicity rate - Chronic resolution rate - Hospitalization frequency (acute and chronic) - Outpatient hospitalization frequency - Mortality rate - Chronicity rate - Rate and duration of: arthralgia, arthritis, fatigue, fever, headache, joint pain, joint swelling, myalgia, nausea, rash, and vomiting | None |
| Study design (S) | - Randomized (and non-randomized) clinical trial - Systematic reviews and meta-analyses **^a^** - Observational studies - Real-world evidence studies | - In vitro studies - Preclinical studies - Reviews - Comments, letters, and editorials - Case studies ^b^ - Case series ^b^ |
| Language | English language | None |
| Time limit | No time limit | None |
| Country | No restriction | None |
| Note: If it is unclear whether a study meets any criterion during the Level 1 screening process, the study will be progressed to full-text screening to confirm its inclusion in the review.  ^a^ Systematic reviews and meta-analyses were included at Level 1 screening, used for the identification of primary studies, and then excluded at Level 2 screening.  ^b^ Studies that reported multiple cases were included after consolidation with multiple screeners | | |
