# Supplementary material for: Clinical outcomes of chikungunya across age groups: A systematic review
Source: PLoS Negl Trop Dis. 2025 Oct 21;19(10):e0013580. doi: 10.1371/journal.pntd.0013580 (PMC12539745; doi:10.1371/journal.pntd.0013580)
Supplement: S2 File — (DOCX) [file pntd.0013580.s002.docx]

**S2 File. Reported prevalence rates of most reported outcomes.**

Figure 1. Rash rates reported for child, adult and elderly populations.

Figure 2. Fever rates reported for child, adult and elderly populations

Figure 3. Headache rates reported for child, adult and elderly populations

Figure 4. Arthralgia rates reported for child, adult and elderly populations. Note: one study reported the incidence of arthralgia and arthritis as one value (65). This outcome (60.00%, N=45) was included both in this graph and in the arthritis graph.

Figure 5. Myalgia rates reported for child, adult and elderly populations

Figure 6. Vomiting rates reported for child, adult and elderly populations

Figure 7. Arthritis rates reported for children, adult and elderly populations. Note: one study reported the incidence of arthralgia and arthritis as one value (65). This outcome (60.00%, N=45) was included both in this graph and in the arthralgia graph.
