# Supplementary material for: Clinical outcomes of chikungunya across age groups: A systematic review
Source: PLoS Negl Trop Dis. 2025 Oct 21;19(10):e0013580. doi: 10.1371/journal.pntd.0013580 (PMC12539745; doi:10.1371/journal.pntd.0013580)
Supplement: S3 File — (DOCX) [file pntd.0013580.s003.docx]

**S3 File. Reported prevalence rates of mortality, hospitalization and chronic disease.**

Figure 8. Mortality rates reported for young, adult and elderly populations. Highlighted in green are newborn cases.

Figure 9. Hospitalization rates reported for young, adult and elderly populations.

Figure 10. Chronic disease rates reported for young, adult and elderly populations.
