# Supplementary material for: Clinical outcomes of chikungunya across age groups: A systematic review
Source: PLoS Negl Trop Dis. 2025 Oct 21;19(10):e0013580. doi: 10.1371/journal.pntd.0013580 (PMC12539745; doi:10.1371/journal.pntd.0013580)
Supplement: S6 Table — (DOCX) [file pntd.0013580.s010.docx]

**S6 Table. Summary of outcomes reported in fewer than 25 studies.**

|  | Children | | | Adults | | | Elderly | | |
| --- | --- | --- | --- | --- | --- | --- | --- | --- | --- |
| Outcomes | **#** | **Min/max reported** | **Reference** | **#** | **Min/max**  **reported** | **Reference** | **#** | **Min/max**  **reported** | **Reference** |
| Acute illness duration | 2 | 2.0 days–5.0 days (median) | (14, 49) | 3 | 2.0 days – 5.5 days (mean) | (66, 67) | - |  |  |
| Arthralgia duration | - |  |  | 1 | 8.2 (days before improvement of symptom) | (68) | - |  |  |
| Arthritis prevalence | 5 | 0.0%–60.0% | (14, 33, 49, 65, 69) | 20 | 0.0%-100.0% | (33, 39, 48, 54, 67, 68, 70-78) | 1 | 12.7% | (37) |
| Duration of chronic disease | - |  |  | 2 | 12.0 months – 185.0 months (minimum, maximum) | (74, 79) | - |  |  |
| Fatigue prevalence | 2 | 0.0%–60.0% | (14, 29) | 9 | 3.0%-100% | (3, 39, 54, 70, 80-83) | - |  |  |
| Fever duration | 2 | 2.0 days (mean)  4.0 days (median) | (84, 85) | 9 | 3.1 days – 9.5 days (mean) | (68, 83, 86-89) | - |  |  |
| Joint pain duration | - |  |  | 3 | 21.3 days – 141.0 days (mean) | (68, 87) | - |  |  |
| Joint pain prevalence | 7 | 29.7%–95.0% | (25, 29, 43, 58, 90-92) | 12 | 36.9%-100% | (3, 32, 44, 68, 89, 93-97) | 1 | 38.3% | (36) |
| Joint Swelling duration | - |  |  | 1 | 51.0 days | (87) | - |  |  |
| Joint swelling prevalence | 5 | 10.0%–49.9% | (14, 25, 29, 92, 98) | 11 | 18.5%-76.5% | (32, 51, 66, 67, 83, 96, 97, 99) | - |  |  |
| Nausea prevalence | 11 | 11.6%– 70.4% | (14, 26, 27, 38, 49, 65, 69, 90, 98, 100, 101) | 16 | 0.9%-76.5% | (45, 46, 48, 51, 54, 66, 75, 77, 97, 102-106) | - |  |  |
| Overall symptomatic disease prevalence | - |  |  | 6 | 100% | (50, 68, 74, 95, 97) | - |  |  |
| *Abbreviations: #, number of times outcome was reported for a population; min, minimum; max, maximum* | | | | | | | | | |
