# Supplementary material for: Clinical outcomes of chikungunya across age groups: A systematic review
Source: PLoS Negl Trop Dis. 2025 Oct 21;19(10):e0013580. doi: 10.1371/journal.pntd.0013580 (PMC12539745; doi:10.1371/journal.pntd.0013580)
Supplement: Attachment — Submitted filename: Response to reviewers.docx [file pntd.0013580.s012.docx]

**Clinical Outcomes of Chikungunya Across Age Groups: A Systematic Review**

Dear reviewers,

On behalf of all authors, I thank the reviewers for taking the time to comment on our manuscript. Below you can find the response to all the comments. In case we had made adaptations in the manuscript, we refer to the lines we adjusted in the version of the manuscript with track changes.

Kind regards,

Judith Bentvelzen

**Reviewers' Comments:**
Reviewer #1 – General comments
The manuscript, entitled 'Clinical Outcomes of Chikungunya Across Age Groups: A Systematic Review', by Judith Bentvelzen et al., is a timely and well-structured systematic review of age-related differences in the clinical outcomes of chikungunya virus (CHIKV) infections. The categorisation of clinical outcomes in children, adults and the elderly fills a significant gap in the literature and is highly relevant to diagnostics, clinical management and public health policy. However, there are four major and one minor issue that should be addressed to strengthen the manuscript's rigour and impact.

- *The authors thank the reviewer for the kind words, thorough review, and helpful feedback. The reviewer’s feedback has been incorporated with the responses described below.*

Reviewer #1 - Major concerns
1. Firstly, the manuscript does not explore potential differences in clinical presentation linked to the infecting CHIKV genotype (e.g. ECSA, Asian or Indian Ocean lineage). Given the evidence that certain genotypes (e.g. IOL) are associated with an increased risk of chronic arthralgia or atypical presentations, this is a significant omission. At the very least, it would be valuable to include a discussion on this topic, even if data could not be extracted from most studies.

- *The authors thank the reviewer for this valuable comment. We acknowledge that chikungunya virus genotype can influence clinical presentation, including the risk of chronic disease. In order to make an age-specific comparison possible, we had to maximize the number of studies eligible for inclusion in our age-stratified analysis and we did not include genotype reporting as a selection criterion. As a result, a stratified analysis by genotype was not feasible. Additionally, given the already substantial heterogeneity in reporting across studies, such a combined stratification was not methodologically feasible. Attempting to do so would have limited comparability of age groups and undermined the validity of the age-related findings that are central in this review. That said, we recognize the importance of viral lineage in shaping disease outcomes and have addressed this limitation explicitly in the revised discussion (lines 460-471), including relevant evidence on genotype-related clinical variation and a recommendation for future research to report and explore genotype data in relation to patient characteristics such as age.*

2. While the review is systematic and descriptive, the presentation remains largely narrative. Key outcome comparisons (e.g. the prevalence of arthralgia or fever across age groups) could benefit from basic quantitative synthesis, such as forest plots or weighted averages, even if formal meta-analysis is precluded by heterogeneity.

- *The authors agree that a more quantitative synthesis can greatly enhance interpretability and add value to systematic reviews. Accordingly, the feasibility of performing a meta-analysis was done, and options for visual quantitative summaries were explored. However, due to the substantial heterogeneity across studies (e.g., population definitions, case criteria, symptom reporting, and age stratifications) a reliable summary using pooled point estimates was not feasible. The authors were concerned that forcing quantitative synthesis under these conditions could lead to misleading interpretations.*

*To provide a sense of the distribution and frequency of key outcomes, the manuscript includes summary figures with reported rates and study sizes in Appendix 5 and 6. While these do not represent formal meta-analyses or weighted averages, they aim to give readers an overview of patterns across studies. Also, a clarification in the Methods section (Lines 98-101) was added, to explain the rationale for not performing quantitative pooling.*

3. The review could address the diagnostic challenges posed by atypical presentations, especially in elderly patients, more effectively. This could explain underreporting or misclassification in older age groups, which has important implications for case definitions and surveillance strategies.

- *To address this important issue in more depth, we have added a dedicated paragraph to the Discussion section (Lines 403–413). This addition elaborates on the clinical relevance of atypical disease presentations, particularly in older adults, and highlights how their misalignment with the current WHO case definition may contribute to both misdiagnosis and underreporting in this population.*

4. While the manuscript acknowledges high heterogeneity, further efforts could be made to analyse the impact of variable diagnostic criteria, symptom definitions or inclusion thresholds (e.g. studies requiring fever for enrolment). Sensitivity analyses or stratification by study quality could strengthen the interpretation of the findings.

- *We thank the reviewer for this constructive suggestion. We considered further stratified synthesis based on inclusion criteria (e.g. fever required vs. not) but ultimately decided not to implement this in the main text. Given the small number of included studies and the limited number within each potential stratum, we judged that such an approach would risk over-fragmenting the synthesis and diluting interpretability. We also aimed to balance comprehensiveness with clarity in the result figures, as these are already visually dense (Appendix 5 and 6).*

*To address the issue of heterogeneity in inclusion criteria and diagnostic definitions, we made efforts to clearly tabulate the population characteristics, inclusion and exclusion criteria for each of the most reported outcomes, as well as for the mortality, hospitalization and chronic disease outcomes (Appendix 7). In line with the reviewer’s suggestion, the study quality has been included in this document. Studies with good and excellent quality were reported throughout the full Results section, for example see lines 176-177 and lines 264-266, to help contextualize the strength and consistency of the evidence. Studies with a low risk of bias are also highlighted in the overview table (Table 1).*

Minor concern:
5. The methods could be clarified to avoid the use of overlapping terms such as 'arthralgia', 'joint pain' and 'arthritis'. Were these harmonised, or were they reported as distinct endpoints?

- *We appreciate the reviewer’s observation. In the included studies, the terms ‘arthralgia’, ‘joint pain’ and ‘arthritis’ were all reported as distinct endpoints and extracted as such. We did not harmonise these terms post hoc, given that in some studies these are considered clinically and methodologically distinct constructs. We have further clarified the distinction and reasoning in the Methods (Lines 78-81), to avoid ambiguity and to ensure transparency regarding how symptom data were handled.*

**Reviewer #2**

This is a welcome paper at a time when two chikungunya vaccines have been licensed and policy makers are still trying to get their heads around the optimal use of such vaccines, which includes age specific use. This paper shows what we already know in some way, but now describes it with more clarity and more data: Chikungunya affects age groups differently: young children and the elderly face higher risks of severe disease, while older children recover faster. Symptom patterns vary by age, highlighting the need for age-specific healthcare strategies and further research.

The authors did a systematic review and used the PRISMA approach.

Introduction:
1. May I suggest that the authors also highlight, within the epidemiological overview, that chikungunya is a common infection among international travellers? To support this, the following references could be considered for inclusion:

Chikungunya infection in returned travellers: results from the geosentinel network, 2005-2020.
Bierbrier R, Javelle E, Norman FF, Chen LH, Bottieau E, Schwartz E, Leder K, Angelo KM, Stoney RJ, Libman M, Hamer DH, Huits R, Connor BA, Simon F, Barkati S; GeoSentinel Network. J Travel Med. 2024 Mar 1;31(2):taae005. doi: 10.1093/jtm/taae005. PMID: 38195993

Chikungunya: risks for travellers.
Simon F, Caumes E, Jelinek T, Lopez-Velez R, Steffen R, Chen LH. J Travel Med. 2023 Apr 5;30(2):taad008. doi: 10.1093/jtm/taad008.

- *The authors agree with this valuable suggestion. Indeed, highlighting the burden of chikungunya among international travellers adds important epidemiological context, particularly in light of global mobility and the associated risks of disease introduction into non-endemic areas. In line with the reviewer’s recommendation, we have revised the Introduction (Lines 8-12) to note that chikungunya is also a well-recognized cause of illness among international travellers. We have cited both of the suggested references (Bierbrier et al., 2024; Simon et al., 2023) to strengthen this point.*

2. In terms to climate change and increased vectorial capacity, maybe the authors could consider adding this reference in addition to the one by Liu/Rocklov:
Climate change and vector-borne diseases: a multi-omics approach of temperature-induced changes in the mosquito.
Bellone R, et al. Failloux AB. J Travel Med. 2023 Jun 23;30(4):taad062. doi: 10.1093/jtm/taad062.

- *We thank the reviewer for this insightful comment. As recommended, we have now included the reference by Bellone et al. (2023) in the Introduction section (Lines 6-8). This addition strengthens the discussion on climate change and its impact on mosquito vectorial capacity, providing further context to the role of environmental changes in the spread of chikungunya and the way that molecular adaptations further allow for the spread of the vector.*

Discussion:
3. While the authors' findings are consistent with previous studies showing that young children and older adults are at highest risk for severe chikungunya outcomes, further discussion is warranted. Given that chikungunya infection is significantly more prevalent in adults—leading to substantial morbidity despite relatively low mortality—the overall DALY (Disability-Adjusted Life Years) burden and socio-economic impact may in fact be greater in this age group than in the smaller populations of the very young and very old. Although the latter may have higher relative mortality, the absolute numbers are comparatively lower. There appears to be a strong focus in the current discourse on prioritizing vaccine use for the very young and the elderly. However, the authors should consider whether this truly represents the most optimal vaccination strategy. A discussion on this point would be valuable, and the authors are encouraged to stimulate further work in the form of economic evaluations, DALY assessments, and modelling studies to guide future policy decisions.

- *We appreciate the reviewer’s constructive input. In line with the suggestion, we have expanded the Discussion (Lines 413-424) to highlight that, although infants and the elderly have higher relative risk of severe outcomes, adults account for the majority of chikungunya cases. Consequently, adults experience a disproportionate share of the overall disease burden, particularly due to the chronic and disabling nature of arthralgia in this group. We have altered the section concerning vaccine prioritization to consider whether targeting the very young and elderly in vaccination truly addresses the full burden of disease. We agree with the reviewer that further health and economic studies are warranted to guide policy makers on the most effective allocation of vaccines.*

4. Regarding vaccines, I would also recommend that the authors expand their reference list to include more literature on chikungunya vaccines, including the following:
From bench to clinic: the development of VLA1553/IXCHIQ, a live-attenuated chikungunya vaccine.
Chen LH, Fritzer A, Hochreiter R, Dubischar K, Meyer S. J Travel Med. 2024 Oct 19;31(7):taae123. doi: 10.1093/jtm/taae123. PMID: 39255380

- *We thank the reviewer for this helpful suggestion. The authors have now included the reference in the Introduction (Lines 41-45). In addition, we have added a paper from Rosso et al. (2024) (PMID: 3934000), a systematic review and meta-analysis on the immunogenicity and safety of chikungunya vaccines. These additions provide a more comprehensive overview of current chikungunya vaccine development, and the value of age-specific findings to allow for effective prevention strategies across age groups.*
